# ICA-based artifact removal diminishes scan site differences in multi-center resting-state fMRI

**DOI:** 10.3389/fnins.2015.00395

**Published:** 2015-10-27

**Authors:** Rogier A. Feis, Stephen M. Smith, Nicola Filippini, Gwenaëlle Douaud, Elise G. P. Dopper, Verena Heise, Aaron J. Trachtenberg, John C. van Swieten, Mark A. van Buchem, Serge A. R. B. Rombouts, Clare E. Mackay

**Affiliations:** ^1^Department of Radiology, Leiden University Medical CentreLeiden, Netherlands; ^2^FMRIB, Nuffield Department of Clinical Neurosciences, Oxford Centre for Functional Magnetic Resonance Imaging of the Brain, University of OxfordOxford, UK; ^3^Department of Psychiatry, University of OxfordOxford, UK; ^4^Department of Neurology, Erasmus Medical CentreRotterdam, Netherlands; ^5^Leiden Institute for Brain and Cognition, Leiden UniversityLeiden, Netherlands; ^6^Institute of Psychology, Leiden UniversityLeiden, Netherlands

**Keywords:** resting-state functional MRI, multi-center analysis, independent component analysis, dual regression, structured noise reduction

## Abstract

Resting-state fMRI (R-fMRI) has shown considerable promise in providing potential biomarkers for diagnosis, prognosis and drug response across a range of diseases. Incorporating R-fMRI into multi-center studies is becoming increasingly popular, imposing technical challenges on data acquisition and analysis, as fMRI data is particularly sensitive to structured noise resulting from hardware, software, and environmental differences. Here, we investigated whether a novel clean up tool for structured noise was capable of reducing center-related R-fMRI differences between healthy subjects. We analyzed three Tesla R-fMRI data from 72 subjects, half of whom were scanned with eyes closed in a Philips Achieva system in The Netherlands, and half of whom were scanned with eyes open in a Siemens Trio system in the UK. After pre-statistical processing and individual Independent Component Analysis (ICA), FMRIB's ICA-based X-noiseifier (FIX) was used to remove noise components from the data. GICA and dual regression were run and non-parametric statistics were used to compare spatial maps between groups before and after applying FIX. Large significant differences were found in all resting-state networks between study sites before using FIX, most of which were reduced to non-significant after applying FIX. The between-center difference in the medial/primary visual network, presumably reflecting a between-center difference in protocol, remained statistically significant. FIX helps facilitate multi-center R-fMRI research by diminishing structured noise from R-fMRI data. In doing so, it improves combination of existing data from different centers in new settings and comparison of rare diseases and risk genes for which adequate sample size remains a challenge.

## Introduction

Resting-state functional Magnetic Resonance Imaging (R-fMRI) has become an important tool in neuroimaging research to examine Resting-State Networks (RSNs) in normal brains, during the aging process and in various neurological disorders (Greicius et al., 2003; Fox et al., 2005; De Luca et al., 2006; Fox and Raichle, 2007; Littow et al., 2010). One of the techniques used for this purpose is Independent Component Analysis (ICA)—a data-driven technique that facilitates comparison of functional networks in the brain without requiring a priori selected seed regions (Beckmann and Smith, 2004).

(R-)FMRI research has certain challenges, such as problems regarding sample size in clinical and at-risk populations. Multi-center analysis may help to solve these limitations, but has been shown to be difficult to perform for (R-)fMRI. Specifically, differences between groups may not always be attributable to the feature of interest, such as disease or gene carrier status, but may also be secondary to scanner hardware differences (manufacturer, head-coil), software differences (filters, k-space acquisition method, scan parameters), and environmental differences (radio-frequency noise) (Casey et al., 1998; Zivadinov and Cox, 2008). Confounding center effects also manifest as noise in multi-center analysis, reducing power.

Whilst several studies have investigated and provided guidelines and recommendations for these difficulties for fMRI (Zou et al., 2005; Costafreda et al., 2007; Friedman et al., 2008; Wegner et al., 2008; Zivadinov and Cox, 2008; Glover et al., 2012), multi-center research using R-fMRI is still an underdeveloped field. Long et al. (2008) were able to cross-validate the Default Mode Network (DMN) in a multi-center study even though scanner parameters were not controlled. Biswal et al. (2010) demonstrated that functional connectivity has a universal architecture in an extensive study with 1414 subjects. However, they also found many differences due to center-related variability. As data sharing becomes more important in imaging research [e.g., the Genetic Frontotemporal dementia Initiative (GenFI), Rohrer et al., 2013; 1000 Functional Connectome Project, Biswal et al., 2010; ADHD 200 Consortium dataset (The ADHD-200 Consortium, 2012); and Autism Brain Imaging Data Exchange (ABIDE), Di Martino et al., 2014], methods for reducing scan site differences must be developed.

In the current study, a novel tool for the clean up of structured noise-components from ICA was used to study whether R-fMRI data from different scan sites become more comparable in a multi-center analysis. The Functional Magnetic Resonance Imaging of the Brain Centre's (FMRIB's) ICA-based X-noiseifier (FIX) is a plug-in to FMRIB's Software Library (FSL) that is able to automatically classify and remove structured noise-components (e.g., motion-effects, scanner artifacts, (non-neuronal) physiological noise, etc.) from R-fMRI data, once it has been trained through hand-classifications (Griffanti et al., 2014; Salimi-Khorshidi et al., 2014). FIX has been used before to clean up structured noise in order to heighten the quality of R-fMRI data (Salimi-Khorshidi et al., 2014), but this is the first time FIX is used to diminish scanner differences in a multi-center study.

## Methods

### Participants

In this study, MRI data was included from subjects scanned at the Leiden University Medical Centre (LUMC) and from subjects scanned at the University of Oxford Centre for Clinical Magnetic Resonance Research (OCMR). The LUMC data (referred to in this article as the “Dutch” sample) consisted of 36 subjects from the control group of an earlier R-fMRI study investigating the effect of *microtubule-associated protein tau* (*MAPT*) and *progranulin* (*GRN*), risk genes for Frontotemporal Dementia (FTD), on the brain (Dopper et al., 2013). The OCMR data (referred to in this article as the “English” sample) consisted of 36 subjects from control groups of earlier R-fMRI studies investigating the effect of *apolipoprotein E* ε*4* (*APOE* ε*4*), a risk gene for Alzheimer's Disease (AD), on the brain (Filippini et al., 2009, 2011; Heise et al., 2011; Trachtenberg et al., 2012a,b).

The English subjects were selected from a larger cohort in order to match the Dutch subjects in age, gender and sample size.

For a detailed description of the recruitment protocols, see Dopper et al. (2013) for the Dutch data and Filippini et al. (2009), Filippini et al. (2011), and Trachtenberg et al. (2012b) for the English data. In short, 36 *MAPT*- and *GRN*-non-carriers were selected from a pool of 160 healthy first-degree relatives of FTD patients with either a *MAPT*- or *GRN*-mutation. It is assumed that the non-carriers from these families have the same risk for dementia as the general population. Thirty-six *APOE* ε*4*-non-carriers, scanned at the OCMR, were selected from the general population and the data from most (30/36) were reported in previous studies (Filippini et al., 2009, 2011; Trachtenberg et al., 2012b).

Pre-scan exclusion criteria included MRI contraindications, history of drug abuse, and current or past neurologic or psychiatric disorders for the Dutch sample, and head injury, substance abuse (including alcohol), corticosteroid therapy, youth diabetes therapy, memory complaints, and current or past neurologic or psychiatric disorders for the English sample.

All participants provided written informed consent, and ethical approval for data acquisition was obtained from National Research Ethics Service Committee South Central—Oxford C (Oxford data) and the Medical Ethical Committees in Rotterdam and Leiden (Leiden data).

### Image acquisition

LUMC scans were acquired using a Philips 3.0T Achieva MRI scanner with an 8-channel SENSE head coil. OCMR scans were acquired using a Siemens 3.0T Trio scanner with a 12-channel head coil. Participants were instructed to keep their eyes closed (LUMC) or open (OCMR), to think of nothing in particular (OCMR) and to remain awake. The scan parameters used for the high-resolution 3D anatomical T1-weighted and for the R-fMRI T2*-weighted images are shown in Table 1.

**Table 1:** **Structural and functional scan parameters per scan site**.

**Parameters**	**Structural**		**Resting-state**	
	**LUMC**	**OCMR^a^**	**LUMC**	**OCMR**
TR	9.8 ms	2040 ms	2200 ms	2000 ms
TE	4.6 ms	4.7 ms	30 ms	28 ms
Flip angle	8°	8°	80°	89°
Number of slices/FOV	140 slices	FOV = 192 cm^2^	–	–
Number of axial slices	–	–	38	34
Number of volumes	–	–	200	180
Voxel size	0.88 × 0.88 × 1.20 mm	1 × 1 × 1 mm	2.75 × 2.75 × 2.75 mm + 10% interslice gap	3 × 3 × 3.5 mm
Total scan time	5 min	6 min	8 min	6 min

*FOV, field of view; LUMC, Leiden University Medical Centre; OCMR, Oxford Centre for Clinical Magnetic Resonance Research; TE, echo time; TR, repetition time*.

a *Structural scanning at OCMR was done using a magnetization-prepared rapid gradient echo sequence (MPRAGE)*.

### Image analysis

FSL (http://www.fmrib.ox.ac.uk/fsl) tools were used for all data analyses (Smith et al., 2004; Woolrich et al., 2009; Jenkinson et al., 2012).

#### Pre-statistical processing

Individual pre-processing included motion correction (Jenkinson et al., 2002), brain-extraction (Smith et al., 2002), and spatial smoothing using a Gaussian kernel of 6 mm full-width-at-half-maximum (FWHM). 4D grand-mean scaling and high-pass temporal filtering corresponding to a period of 150 s (0.007 Hz) were performed. FMRI volumes were registered to MNI152 standard space (Montreal Neurologic Institute average T1-weighted image created from 152 normal subjects' T1 scans). Boundary-Based Registration (Jenkinson and Smith, 2001; Jenkinson et al., 2002; Greve and Fischl, 2009) was used to register each individual's echo-planar imaging (EPI) volumes onto their respective high-resolution T1-weighted structural images. T1-weighted structural scans were aligned to MNI152 standard space using non-linear image registration (Anderson et al., 2007; Jenkinson et al., 2012). The resulting registration matrices were then used to register the EPI volumes onto MNI152 standard space. Individual ICA was carried out and voxel-by-voxel intensity normalization was performed manually, dividing each voxel by its mean value across time and multiplying by 10,000.

#### FIX

Network components obtained from the individual ICA were visually judged and were labeled as signal, noise, or unknown for 12 subjects from each group. Manual classification was done by looking, firstly, at their spatial maps (typically thresholded abs(Z) > 2.3), then at the temporal power spectrum and lastly at their time-series. Unthresholded spatial maps were examined when necessary (Salimi-Khorshidi et al., 2014).

Using these classifications, the FIX classifier was trained and a training file was created. As described by Salimi-Khorshidi et al. (2014), FIX uses over 180 features, capturing components' spatial and temporal characteristics, which are fed into a multi-level classifier (built around several different classifiers). Temporal features include autoregressive properties, distributional properties, jump amplitudes, the Fourier transform, and the time series' correlation with GM-, WM, CSF-, and head motion-derived time series. Spatial features include clusters' sizes and spatial distribution, voxel intensity information indicating whether voxels are GM or (e.g.,) blood vessels, percent on brain boundary, hand-created mask-based features for components that have signal-like spatiotemporal characteristics (such as sagittal sinus, CSF, and WM) and other spatial features such as spatial smoothness.

Next, a leave-one-out test was run in order to control the quality of the classifier algorithm by estimating the level of agreement of the hand-labeled classifications and the classifier's classifications. The accordance was measured as a true-positive rate (TPR), a true-negative rate (TNR) and a composite measure ((3 · TPR + TNR)/4) for a range of thresholds (used to determine the binary classification of components since FIX's output is probabilistic). After checking the TPR, TNR and the composite measure, the optimal threshold (i.e., 20) was chosen and the classifier was applied to all subjects' data using this threshold in order to classify and remove the structured noise components from the data (Griffanti et al., 2014; Salimi-Khorshidi et al., 2014).

#### GICA

After pre-statistical processing and FIX, three group-level ICA (GICA) analyses were run using MELODIC. In order to qualitatively compare FIX's effect on GICA components, GICA was run on combined English and Dutch data before application of FIX (GICA-1) and on combined English and Dutch data after application of FIX (GICA-2). For statistical analysis of FIX's effect on the multi-center differences, GICA was carried out on all data combined (GICA-3). Consequently, the data used for this analysis (GICA-3) contained four subgroups: Dutch subjects with and without use of FIX, and English subjects with and without use of FIX. R-fMRI data were temporally concatenated across individuals to create a single 4D data set. The data were whitened and principle component analysis was used to project the data into a 25-dimensional subspace, matching many previous R-fMRI studies (Filippini et al., 2009; Smith et al., 2009; Cocozza et al., 2015; Gaudio et al., 2015). By optimizing for non-Gaussian source estimates through a fixed-point iteration technique, we obtained component maps (Hyvärinen, 1999). After transforming the component maps to Z-maps, Gaussian/Gamma Mixture Models were fitted to them in order to obtain 25 independent spatial maps defining functional connectivity patterns across the participants (Beckmann and Smith, 2004). The GICA-derived spatial maps were then judged by eye and divided into RSN and noise components.

#### Dual regression

Analysis of group differences was performed using FSL's dual regression, a regression technique that allows for voxel-wise comparisons of R-fMRI (Filippini et al., 2009; Veer et al., 2010). All spatial maps derived from GICA-3 (using English and Dutch FIX and non-FIX data) were regressed against each individual's pre-processed R-fMRI data, resulting in a time-course for each component and subject. The produced time-courses were regressed against the same individual's pre-processed R-fMRI data, resulting in subject-specific spatial maps for parameter estimates (PEs) and Z-stats. GICA noise component maps were disregarded and RSN component maps were collected across subjects into 4D files (one per ICA component, with the fourth dimension being subject identification) and were tested voxel-wise for statistically significant differences between groups. We used a General Linear Model (GLM) equivalent to two-sample *t*-tests to test the PE- and Z-stat-driven spatial maps for differences between Dutch and English groups before use of FIX, differences between Dutch and English groups after use of FIX, and the interaction between the use of FIX and group differences (by comparing the differences before and after use of FIX to each other). Age and years of education were added to the analysis as confound regressors. Non-paramatric permutation-based testing was done by running 5000 random permutations using the randomize algorithm, a tool based on the Freedman-Lane methods within FSL (Winkler et al., 2014). Afterwards, threshold-free cluster enhancement (TFCE), a method for finding clusters in data without defining clusters in a binary way, was applied (Smith and Nichols, 2009), and a family-wise error-corrected cluster significant threshold of *p* < 0.05 was used. In a more qualitative approach, non-family-wise error-corrected results and raw t-stat maps were also investigated.

#### Result masking

In order to fully appreciate the impact that FIX has on the data, results of the differences between groups for all components were thresholded, binarized, and merged. The resulting imaging volumes display the total number significant voxels for all different components together, with color variation showing the number of components with significant change in each voxel.

### Statistical analysis

Statistics of non-imaging variables were performed using SPSS version 20 (SPSS, Chicago, IL). Demographic variables were tested using independent samples *t*-tests for continuous variables and χ^2^-tests for categorical variables.

## Results

### Sample demographics

Demographic information for the Dutch and English subjects is shown in Table 2. Age and gender were matched across groups.

**Table 2:** **Participant demographics^a^**.

	**OCMR (*n* = 36)**	**LUMC (*n* = 36)**	***p*-value**
Age, y^a^	49.9 (11.5)	49.8 (11.3)	0.943
Gender, % Female	52.8	50.0	1.000
Education, y^a^	16.6 (3.2)	12.6 (2.9)	< 0.001^*^

*LUMC, Leiden University Medical Centre; OCMR, Oxford Centre for Clinical Magnetic Resonance Research*.

a Values denote mean (SD);

* *statistically significant; scores of education level in years were missing for two individuals (both LUMC subjects)*.

### Individual ICA and FIX

Table 3 shows the number of extracted independent components by individual ICA for each group (OCMR and LUMC), as well as the number of components classified as noise and RSN by FIX. Significantly more independent components were extracted from Dutch data, compared to English data. Also, significantly more components from Dutch data were classified as noise by FIX. The number of components classified as RSNs by FIX was not found to be different between groups.

**Table 3:** **FIX classifications^a^**.

	**OCMR (*n* = 36)**	**LUMC (*n* = 36)**	***p*-value**
ICs^a^	36.1 (4.8)	44.3 (7.9)	< 0.001^*^
Noise ICs^a^	23.6 (3.9)	31.8 (8.2)	< 0.001^*^
RSN ICs^a^	12.6 (3.0)	12.7 (3.0)	0.875

*IC, independent component; LUMC, Leiden University Medical Centre; OCMR, Oxford Centre for Clinical Magnetic Resonance Research*.

a Values denote mean (SD);

* *statistically significant*.

### GICA

**Figure 1:**
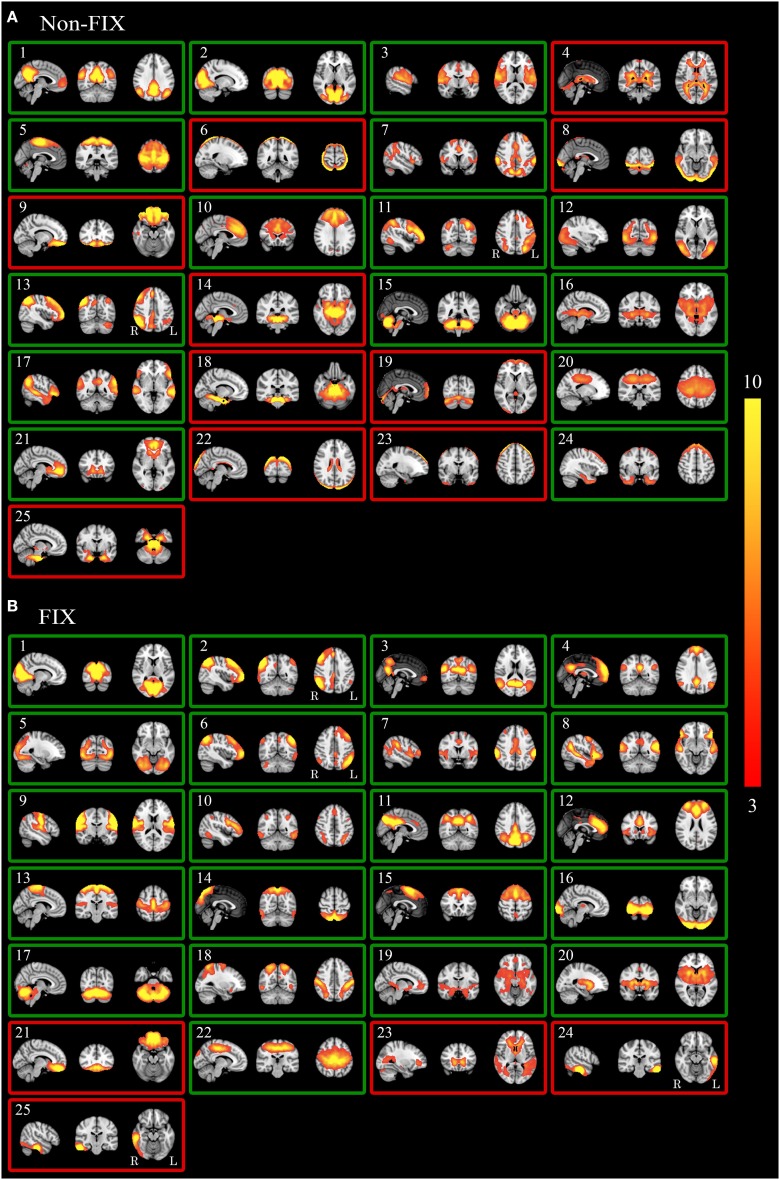
**GICA spatial maps before and after FIX**. Maps illustrate the 25 GICA networks' most informative orthogonal slices before (**A**, GICA-1) and after (**B**, GICA-2) applying FIX. Green frames indicate RSNs; red frames indicate noise networks. Color bar represents Z-scores. *GICA*, Group-level Independent Component Analysis.

Figure 1 shows spatial maps derived from GICA for data before (GICA-1, Figure 1A) and after (GICA-2, Figure 1B) application of FIX (numbers in text correspond to numbers in Figure). RSN components are shown with a green frame, whereas noise components are shown with a red frame. FIX's effect on GICA seems to be two-fold: some noise components are eliminated (i.e., motion artifacts [*1A*: numbers 6, 22, 23] and brain stem/vascular artifacts [*1A*: numbers 14, 18, 25], sagittal sinus artifact [*1A*: numbers 8, 19]) and others are “pushed back” (i.e., have a higher number after the use of FIX: WM [*1A*: number 4, *1B*: number 23] and frontal sinus susceptibility noise [*1A*: number 9, *1B*: number 21]). Both observations rely on the same mechanism: FIX removes variance explained by noise components from the data. As MELODIC shows components in order of decreasing explained variance, the removal of variance explained by noise components results in higher component numbers or even exclusion.

**Figure 2:**
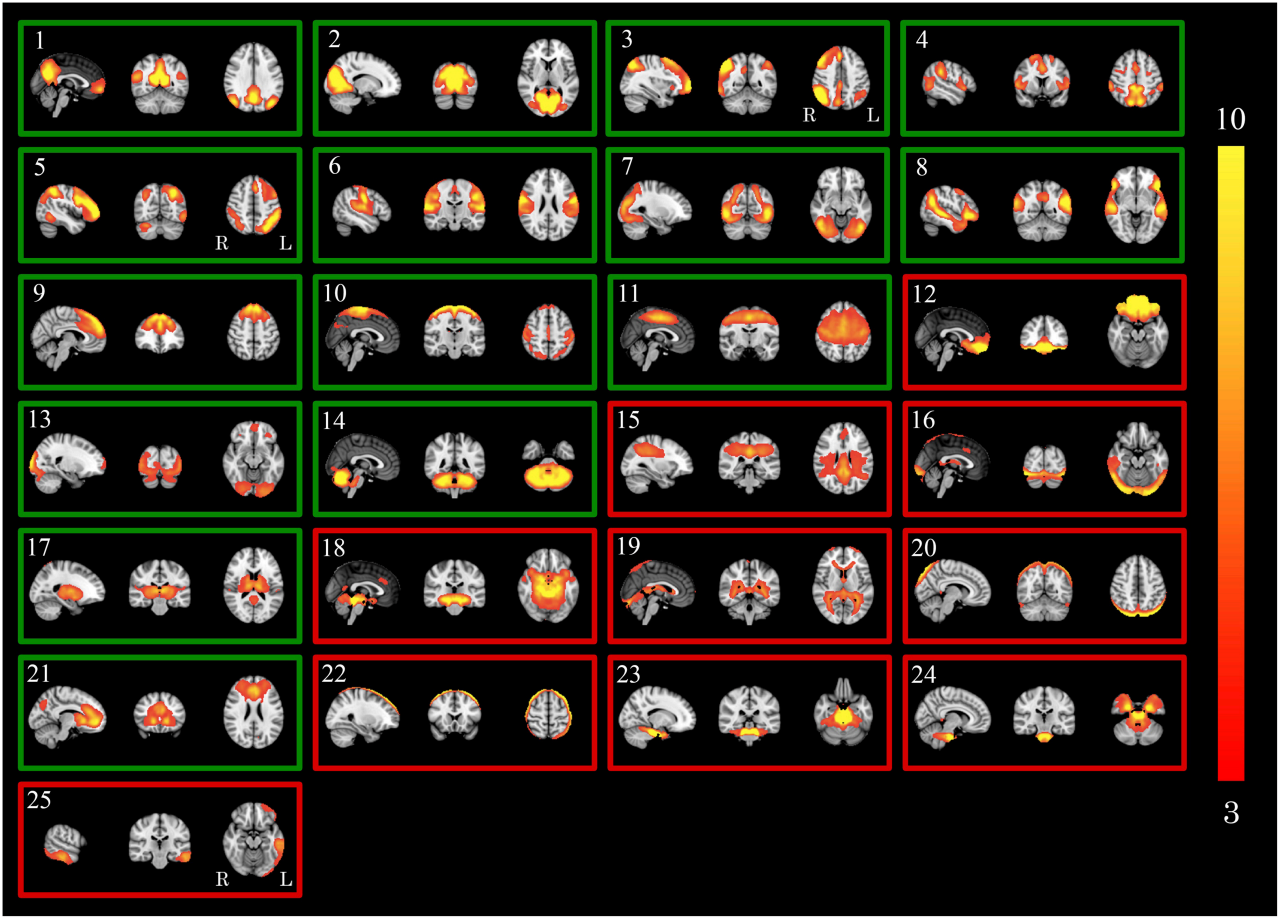
**GICA spatial maps for statistical analysis**. Maps illustrate the 25 GICA networks' most informative orthogonal slices of data before and after applying FIX combined (GICA-3). Green frames indicate RSNs; red frames indicate noise networks. Color bar represents Z-scores. *GICA*, Group-level Independent Component Analysis.

Spatial maps that were used as spatial regressors for dual regression (GICA-3) are shown in Figure 2. Identified RSNs were the DMN [1], primary/medial [2], and lateral [7, 13] visual networks, lateralized higher order cognitive networks involved with working memory [3, 5], a network showing the dorsal attention network combined with the salience network [4], the auditory network [6], a network combining features of the DMN and the ventral stream [8], the executive control network [9], networks that describe different parts of the sensorimotor network [10,11], cerebellar network [14], a network describing the basal ganglia [17] and a network showing frontal DMN features as well as features from the executive control network [21].

### Dual regression

**Figure 3:**
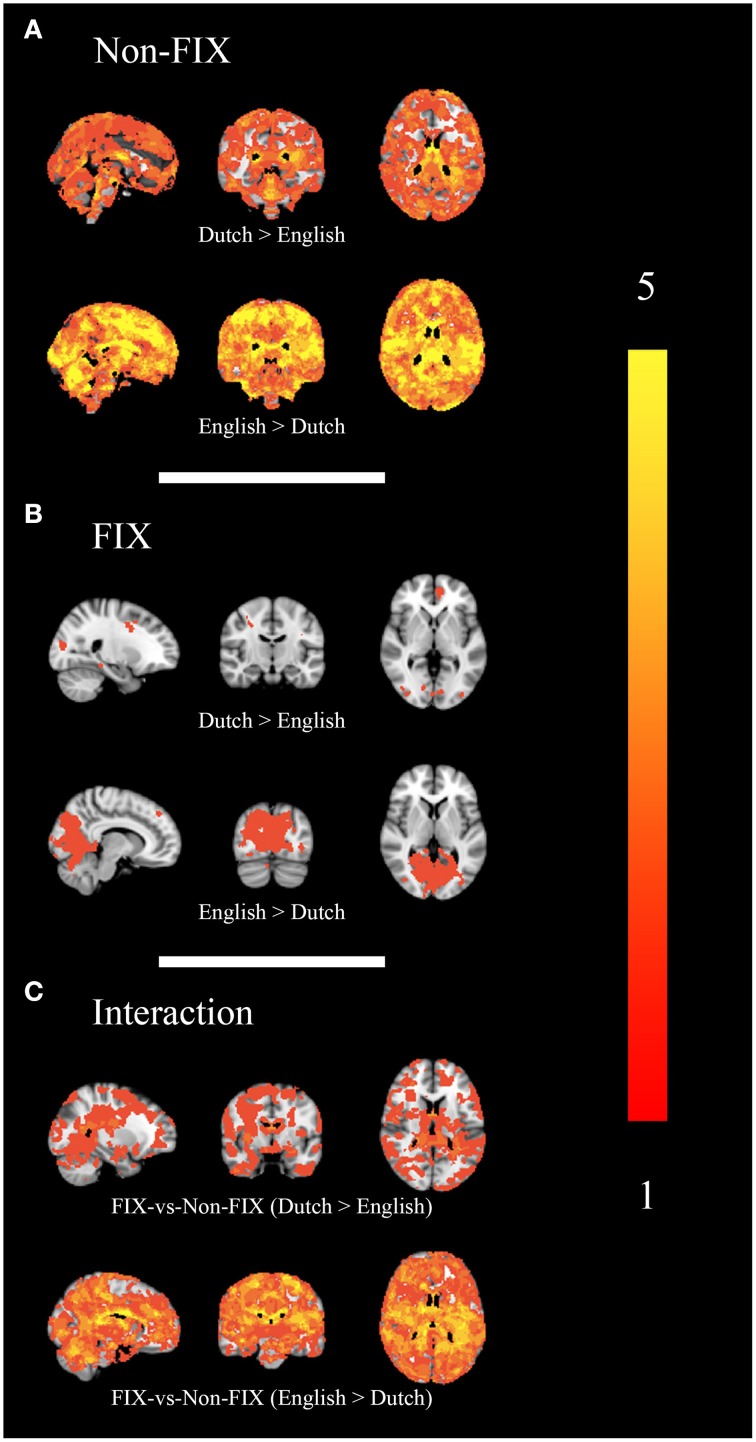
**Combined group differences**. Maps show statistically significant (*p* < 0.05) differences between groups: without the use of FIX **(A)**, after the use of FIX **(B)** and the interaction between FIX and group differences **(C)** in all (15) RSNs combined. Color bar represents the number of significantly differing networks.

All RSNs' combined results based on PE-driven spatial maps are shown for family-wise error-corrected group differences before the use of FIX (Figure 3A), group differences after the use FIX (Figure 3B) and for the interaction between applying FIX and group differences (Figure 3C). Dual regression results for each RSN are shown separately in Supplemental Figure 1 (numbers in Supplemental Figure 1 correspond with numbers in Figure 2).

Before the use of FIX, large areas of statistically significant differences were shown in all (15) RSNs. After applying FIX, the size, and number of areas with significant differences between groups was strongly reduced: only 7/15 RSNs showed statistically significant differences and the number of significantly different voxels was reduced by 98%. The RSN with the largest area of significant differences after using FIX was the primary/medial visual network (PVN), containing 85% of all significantly different voxels after applying FIX. This network is associated with a difference in scan protocol (eyes open vs. closed) and showed greater activation in English than in Dutch subjects.

The interaction between the use of FIX and site-differences was significant in 13/15 RSNs.

Dual regression results based on Z-stat-driven spatial maps were similar on visual inspection.

Additionally, for a more qualitative view of the results, Supplemental Figure 2 shows dual regression results without family-wise error-correction for each component. Another point of view on FIX's effect is offered in Supplemental Figure 3, demonstrating a reduction in raw t-stats for group differences in each component after applying FIX.

## Discussion

In this study, FIX was found to be helpful in the comparison of multi-center R-fMRI data. FIX significantly reduces structured noise resulting from hardware, software, and environmental differences in a multi-center group comparison, as demonstrated by Figure 3 and Supplemental Figures 1, 2. Additionally, Supplemental Figure 3 shows an intra- and inter-component reduction in raw t-stat variability after applying FIX. The significant interaction between the application of FIX and group differences (Figure 3 and Supplemental Figure 1) shows that site-differences are not just pushed below significance threshold, but are significantly changed by applying FIX. Importantly, the remaining differences between sites after FIX (Figure 3 and Supplemental Figure 1) are primarily confined to the primary/medial visual cortex, which reflects differences in experimental design (Dutch participants had eyes closed, whereas English participants had eyes open). This implies that FIX removes structured noise, but retains physiologically driven differences.

Dual regression is usually run using PE-driven spatial maps; alternatively, Z-stat-driven spatial maps can be used. Our results using PE- and Z-stat-driven spatial maps were similar on visual inspection, suggesting that the use of FIX is of value for both types of analysis. However, in order to assess whether FIX works better for either one, a more specific analysis is required.

Structured noise in fMRI has various origins: hardware differences (e.g., scanner manufacturer, type of head coil), software differences (filters, k-space acquisition methods and scan parameters) and radio-frequency noise (Casey et al., 1998; Zivadinov and Cox, 2008). As demonstrated in Figure 3 and Supplemental Figures 1–3, FIX helps to deal with noise from these origins, inasmuch as they present themselves as separate noise components in individual subjects' ICA. Still, it cannot account for all potential between-site differences. For example, it cannot deal with differences that present themselves within RSN components such as differences in sensitivity to RSNs based on hardware configurations or RSN spatial variability relating to head coils. However, due to the nature of ICA, the most striking differences caused by structured noise are presented as separate noise components. Therefore, intra-component variability is much smaller than inter-component variability, implying that the scope of this drawback is altogether limited. Also, FIX cannot account for differences in the magnitude of the BOLD effect. Voxel-wise intensity normalization may help to reduce this problem, but site-wise confound regressors should be used, when they do not correlate with the regressors of interest.

Although eyes-open and eyes-closed differences cannot be mathematically disentangled from site/scanner differences, we suggest that the remaining differences in the PVN after using FIX are a manifestation of this protocol discrepancy since the differences in all other networks are substantially reduced. Recently, a number of studies have investigated the effect of eyes-open vs. eyes-closed conditions on functional connectivity without yet reaching a clear consensus. For example, reduced activation (Feige et al., 2005), amplitude of low frequency fluctuations (ALFF, Yang et al., 2007; Yan et al., 2009; Liu et al., 2013; Liang et al., 2014; Yuan et al., 2014) and regional homogeneity (Liu et al., 2013) have all been reported in eyes-closed relative to eyes-open conditions. Conversely, other studies showed higher BOLD response (McAvoy et al., 2008) and higher mean ALFF (Jao et al., 2013) for the eyes-closed condition, or no difference in seed-based correlations (Patriat et al., 2013). Aside from these local changes in functional connectivity between conditions, Jao et al. discovered that the mean ALFF of the whole brain was greater in eyes-closed vs. eyes-open conditions (Jao et al., 2013). Some of these studies also reported functional connectivity differences in other networks than the PVN, including the sensorimotor, default mode, and auditory networks. The family-wise error-corrected changes found in this study in non-PVN networks were small, scattered, and generally did not follow the IC's anatomy closely. Therefore, it is difficult to infer if these changes are due to the experimental design or if they reflect a small quantity of remaining noise. The changes we found in IC 2 are extensive and follow the PVN anatomy well. As it is unlikely that false positive results or leftover noise would take this form and since similar PVN differences between eyes-open and eyes-closed conditions have been described in R-fMRI multiple times before, it seems reasonable to assume that this effect is due to reported differences in experimental design.

Whilst groups were matched for age and sex, there was a significant difference in level of education. This may be attributable to the recruitment protocols. The English recruitment protocol selected subjects from the general population near Oxford, a relatively highly educated region (Filippini et al., 2009, 2011; Trachtenberg et al., 2012b), whereas the Dutch sample was recruited from known FTD families (Dopper et al., 2013). In order to account for this, demeaned education values were added to the GLM as a regressor of no interest.

Previous studies on multi-center fMRI primarily focused on data collected using standardized protocols. Glover et al. (2012) argue that hardware, software, and procedural aspects should be carefully matched and managed in order to successfully perform multi-center fMRI research. Zivadinov and Cox (2008) suggest the use of quality assurance methods and careful subject selection and matching across centers—such as used by Wegner et al. (2008)—in order to control for scan site by adding center as covariate in the analysis. Whereas these recommendations are naturally important for the correct set-up of a new multi-center study, our results suggest reanalysis of existing non-standardized R-fMRI data may also be possible across sites. Additionally, although it would be interesting to see how these different sources of structured noise are dealt with individually by FIX (whilst controlling for the others), this study importantly shows that even with more of these problems present simultaneously, FIX adequately diminishes structured noise.

## Conclusion

Previous studies using FIX have considered the theoretical and practical use of spatial ICA, classifier training and noise detection (Salimi-Khorshidi et al., 2014) and denoising (Griffanti et al., 2014). They showed that FIX is a useful tool for noise clean up and therefore helps in making data more sensitive to changes related to neuronal activity. This study is the first to show FIX's additional value in multi-center R-fMRI analysis. By improving multi-center fMRI research and efficient reanalysis of acquired data, comparisons of rare diseases and at-risk populations will be more efficient and convenient, leading to a better insight in neurological disorders. Furthermore, as free data sharing is an upcoming way to create large R-fMRI datasets (Biswal et al., 2010; The ADHD-200 Consortium, 2012; Di Martino et al., 2014), FIX may be a valuable tool to ensure valid comparison of data acquired at different centers.

### Conflict of interest statement

The authors declare that the research was conducted in the absence of any commercial or financial relationships that could be construed as a potential conflict of interest.

## Supplementary material

Supplemental Figure 1 **Separate FWE-corrected group differences**. Maps show FWE-corrected statistically significant (*p* < 0.05) differences between groups: without the use of FIX **(A)**, after the use of FIX **(B)** and the interaction between FIX and group differences **(C)** for each of 15 RSNs. Color bar represents significance. TFCE, Threshold-free cluster enhancement.

Supplemental Figure 2 **Maps show non-FWE-corrected statistically significant (*p* < 0.05) differences between groups: without the use of FIX (A) and after the use of FIX (B) for each of 15 RSNs**. Color bar represents significance. FWE, family-wise-error; TFCE, Threshold-free cluster enhancement.

Supplemental Figure 3 **Raw t-stats variability**. Graphs show raw t-stats between groups (positive t-stats signify Dutch > English, negative t-stats signify English > Dutch) before and after FIX for each of 15 RSNs on a logarithmic scale.

## Abbreviations

AD: Alzheimer's Disease
ALFF: amplitude of low frequency fluctuations
APOE ε4: apolipoprotein E ε4
BOLD signal: Blood-oxygen-level-dependent signal
DMN: Default Mode Network
EMC: Erasmus Medical Centre
EPI: echo-planar imaging
FIX: FMRIB's ICA-based X-noiseifier
FMRIB: Functional MRI of the Brain Centre
FSL: FMRIB's Software Library
FTD: Frontotemporal Dementia
FWHM: full-width-at-half-maximum
GICA: group-level ICA
GLM: General Linear Model
GRN: progranulin
ICA: Independent Component Analysis
LUMC: Leiden University Medical Centre
MAPT: microtubule-associated protein tau
MELODIC: Multivariate Exploratory Linear Optimized Decomposition into Independent Components
MNI: Montreal Neurologic Institute
OCMR: Oxford Centre for Clinical Magnetic Resonance Research
PE: Parameter Estimate
PVN: primary/medial visual network
R-fMRI: Resting-state functional Magnetic Resonance Imaging
RSN: Resting-State Network
TFCE: Threshold-free cluster enhancement
TNR: true-negative rate
TPR: true-positive rate.
