# Supplementary figures and images for: ICA-based artifact removal diminishes scan site differences in multi-center resting-state fMRI
Source: Front Neurosci. 2015 Oct 27;9:395. doi: 10.3389/fnins.2015.00395 (PMC4621866; doi:10.3389/fnins.2015.00395)

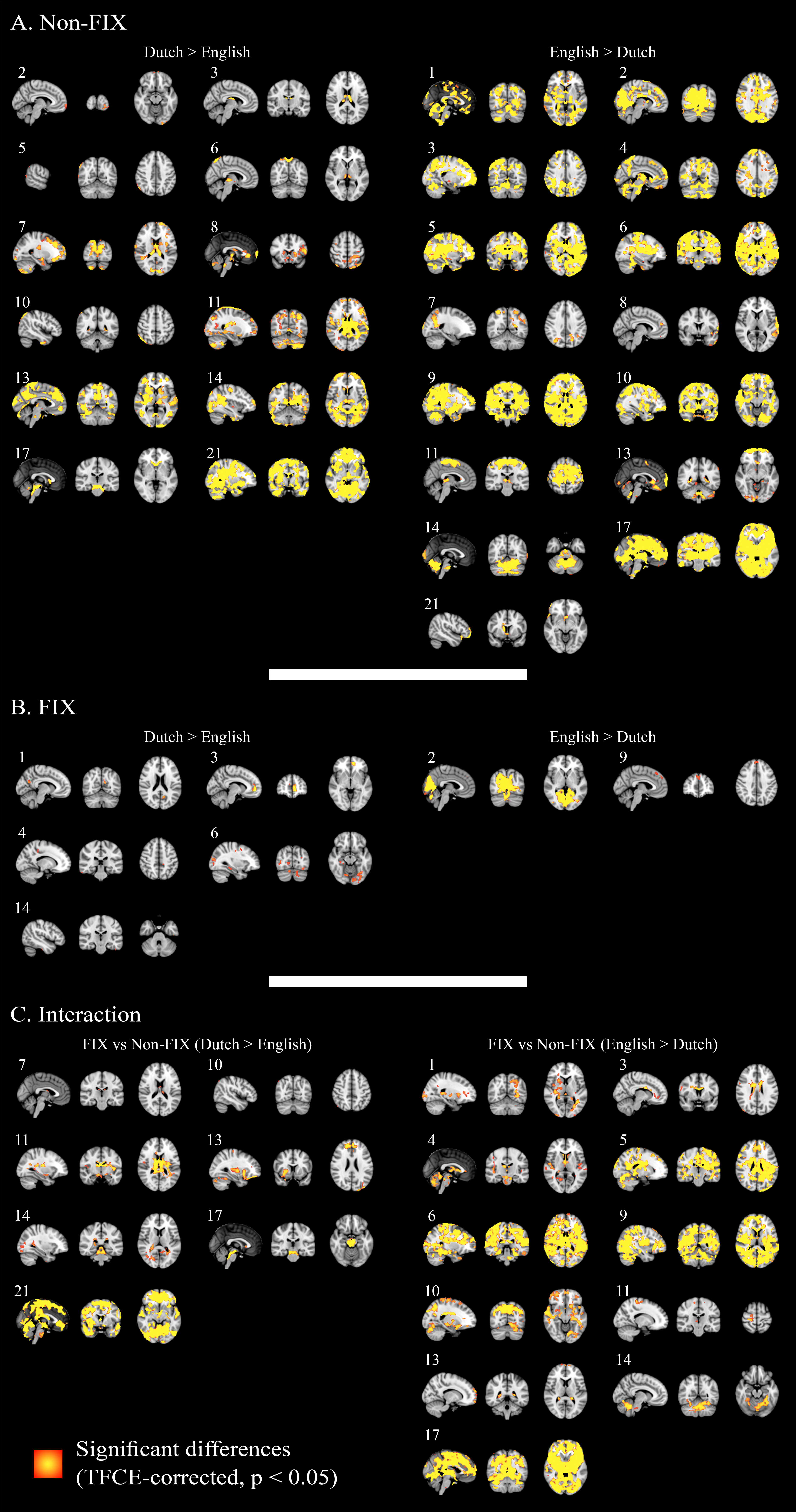

Supplement: Supplemental Figure 1 — Separate FWE-corrected group differences. Maps show FWE-corrected statistically significant (p < 0.05) differences between groups: without the use of FIX (A), after the use of FIX (B) and the interaction between FIX and group differences (C) for each of 15 RSNs. Color bar represents significance. TFCE, Threshold-free cluster enhancement. [file Image1.JPEG]

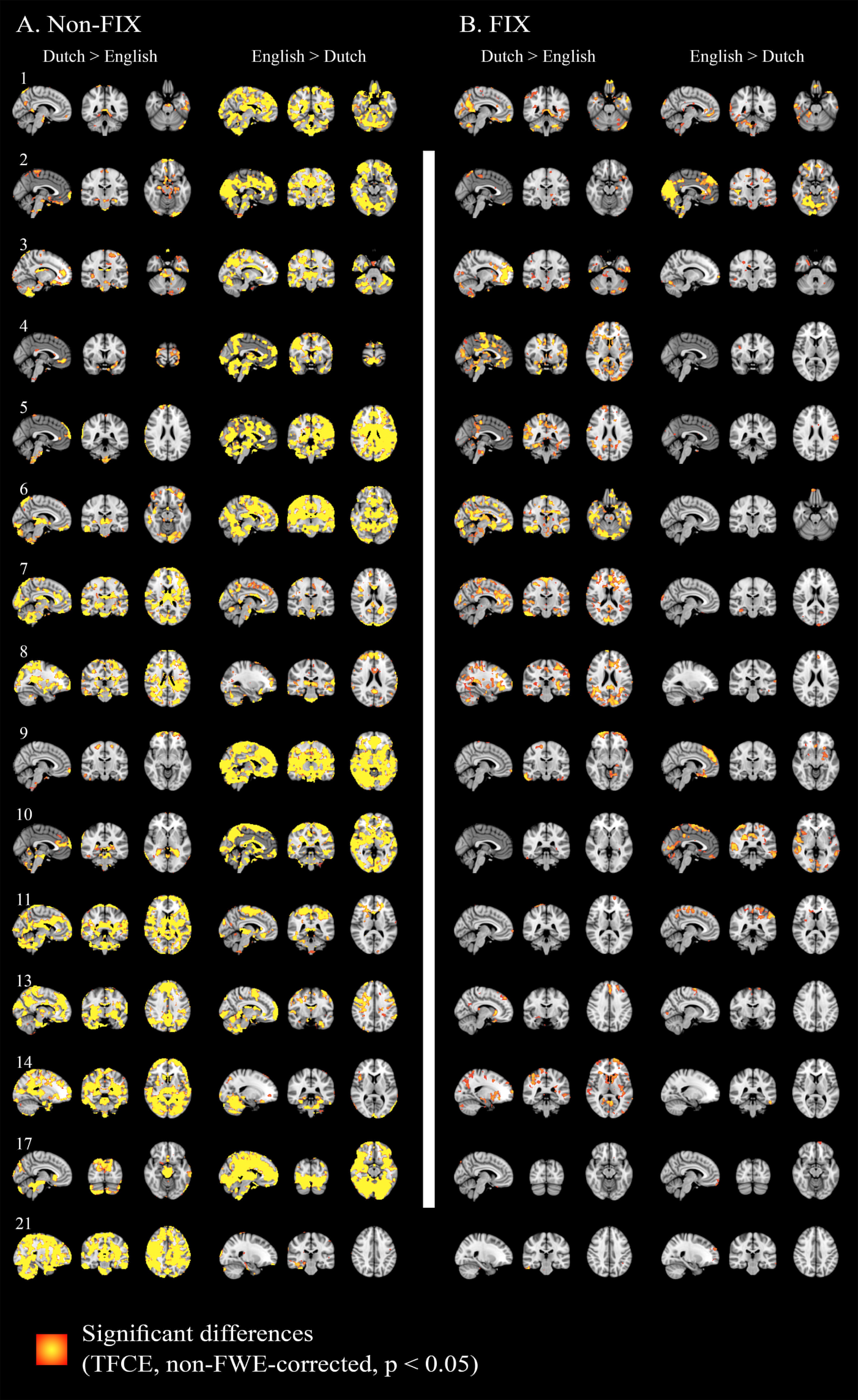

Supplement: Supplemental Figure 2 — Maps show non-FWE-corrected statistically significant (p < 0.05) differences between groups: without the use of FIX (A) and after the use of FIX (B) for each of 15 RSNs. Color bar represents significance. FWE, family-wise-error; TFCE, Threshold-free cluster enhancement. [file Image2.JPEG]

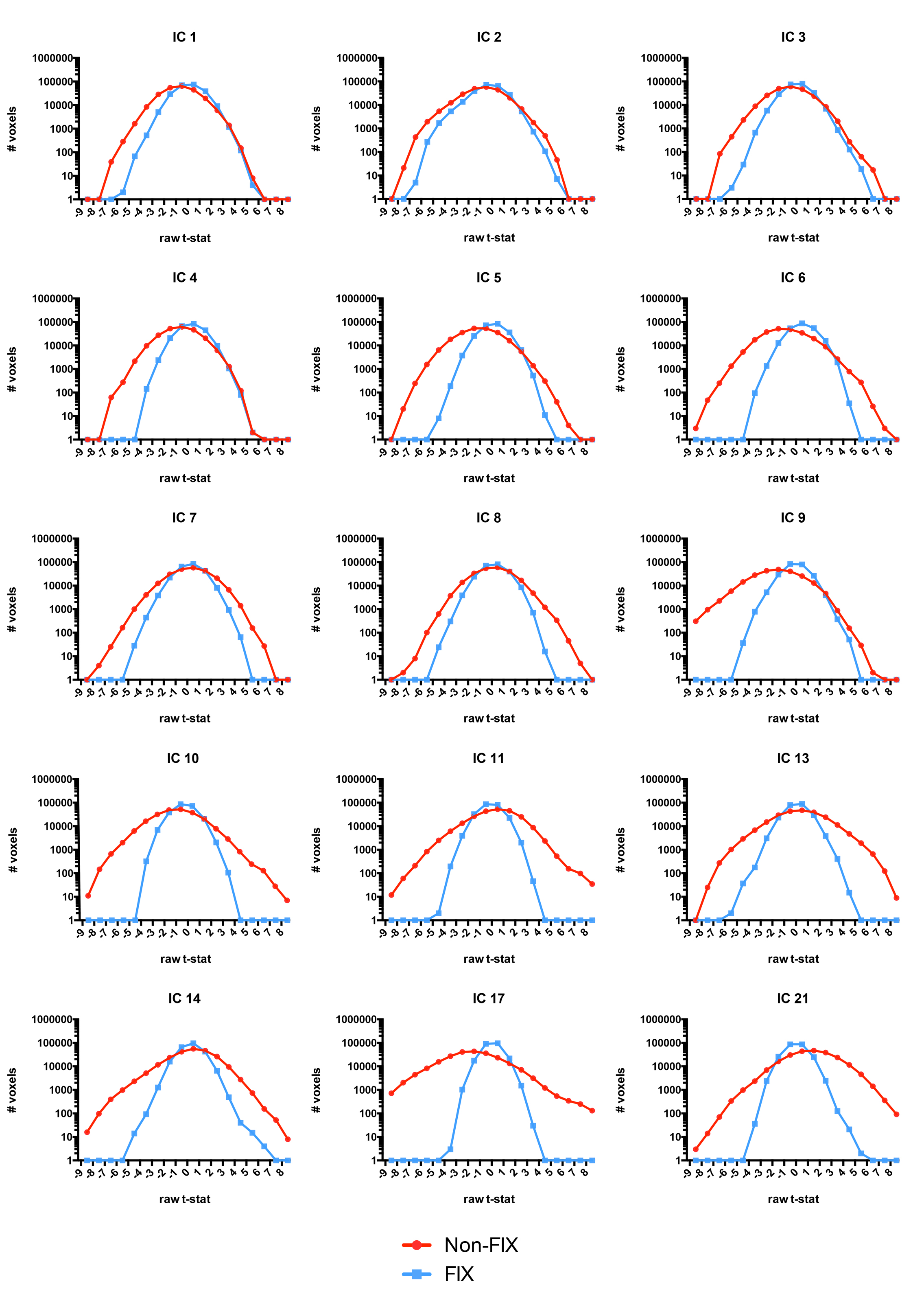

Supplement: Supplemental Figure 3 — Raw t-stats variability. Graphs show raw t-stats between groups (positive t-stats signify Dutch > English, negative t-stats signify English > Dutch) before and after FIX for each of 15 RSNs on a logarithmic scale. [file Image3.JPEG]
